# Methanaminium 3,4,5,6-tetra­chloro-2-(meth­oxy­carbon­yl)benzoate

**DOI:** 10.1107/S1600536811004351

**Published:** 2011-02-12

**Authors:** Jian Li

**Affiliations:** a Department of Chemistry and Chemical Engineering, Weifang University, Weifang 261061, People’s Republic of China

## Abstract

In the crystal structure of the title compound, CH_6_N^+^·C_9_H_3_Cl_4_O_4_^−^, the N atom of the methyl­amine mol­ecule is protonated and hydrogen bonded to the carboxyl group of the 3,4,5,6-tetra­chloro-2-(meth­oxy­carbon­yl)benzoate anion. The anions are linked by the cations *via* inter­molecular N—H⋯O inter­actions into chains extending along the *c* axis.

## Related literature

For related structures, see: Li (2011 ▶); Liang (2008 ▶). 
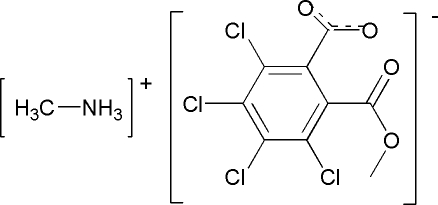


## Experimental

### 

#### Crystal data

CH_6_N^+^·C_9_H_3_Cl_4_O_4_^−^
*M* *_r_* = 348.98
Monoclinic, 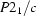

*a* = 14.3138 (13) Å
*b* = 14.2231 (14) Å
*c* = 6.7648 (7) Å
β = 91.021 (1)°
*V* = 1377.0 (2) Å^3^
*Z* = 4
Mo *K*α radiation
μ = 0.87 mm^−1^
*T* = 298 K
0.45 × 0.40 × 0.38 mm

#### Data collection

Bruker SMART CCD area-detector diffractometer
Absorption correction: multi-scan (*SADABS*; Bruker, 1997 ▶) *T*_min_ = 0.697, *T*_max_ = 0.734
6756 measured reflections
2413 independent reflections
1752 reflections with *I* > 2σ(*I*)
*R*_int_ = 0.034

#### Refinement

*R*[*F* ^2^ > 2σ(*F* ^2^)] = 0.034
*wR*(*F* ^2^) = 0.092
*S* = 1.06
2413 reflections
176 parameters
H-atom parameters constrained
Δρ_max_ = 0.27 e Å^−3^
Δρ_min_ = −0.26 e Å^−3^

### 

Data collection: *SMART* (Bruker, 1997 ▶); cell refinement: *SAINT* (Bruker, 1997 ▶); data reduction: *SAINT*; program(s) used to solve structure: *SHELXS97* (Sheldrick, 2008 ▶); program(s) used to refine structure: *SHELXL97* (Sheldrick, 2008 ▶); molecular graphics: *SHELXTL* (Sheldrick, 2008 ▶); software used to prepare material for publication: *SHELXTL*.

## Supplementary Material

Crystal structure: contains datablocks global, I. DOI: 10.1107/S1600536811004351/nc2219sup1.cif

Structure factors: contains datablocks I. DOI: 10.1107/S1600536811004351/nc2219Isup2.hkl

Additional supplementary materials:  crystallographic information; 3D view; checkCIF report

## Figures and Tables

**Table 1:** Hydrogen-bond geometry (Å, °)

*D*—H⋯*A*	*D*—H	H⋯*A*	*D*⋯*A*	*D*—H⋯*A*
N1—H1*A*⋯O3^i^	0.89	1.86	2.742 (3)	173
N1—H1*B*⋯O4^ii^	0.89	1.93	2.794 (3)	163
N1—H1*C*⋯O4	0.89	1.92	2.797 (3)	170

Symmetry codes: (i) 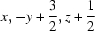; (ii) 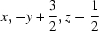.

**Fig. 1.**
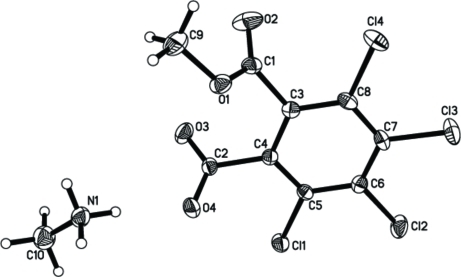
The molecular structure of the title compound with labelling and 30% probability ellipsoids.

**Fig. 2.**
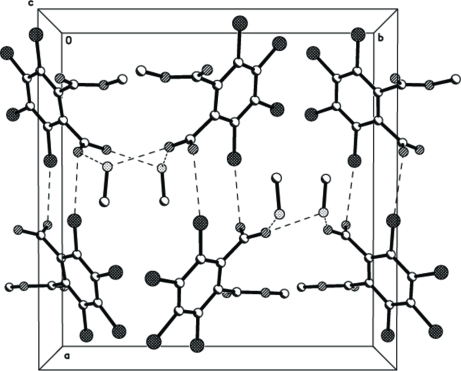
Crystal structure of the title compound viewed along the c axis. Hydrogen bonding and Cl···O interactions are shown as dashed lines.

## supplementary crystallographic information

### Comment

Crystals of the title compound were obtained by accident by the reaction of 2-(methoxycarbonyl)-3,4,5,6-tetrachlorobenzoic acid and methanamine in methanol. To identify the product of this reaction a single crystal structure analysis was performed.

The asymmetric unit of the title compound (I) contains one methylammonium cation and one 2-(methoxycarbonyl)-3,4,5,6-tetrachlorobenzoate anion (Fig. 1). The bond lengths and angles are in agreement with those in ethylammonium 2-(methoxycarbonyl)-3,4,5,6-tetrabromobenzoate methanol solvate (Li, 2011) and in ethane-1,2-diammonium bis(2-(methoxycarbonyl)-3,4,5,6-tetrabromobenzoate) methanol solvate (Liang, 2008). In the crystal structure the cations and anions are connected by intermolecular N—H···O hydrogen bonding into chains that elongate in the direction of the crystallographic c-axis (Fig. 2). Moreover, short distances between chlorine and oxygen atoms are found indicating for intermolecular Cl—O interactions (Fig. 2).

### Experimental

A mixture of 2-(methoxycarbonyl)-3,4,5,6-tetrachlorobenzoic acid (2.86 g, 0.01 mol) and methanol (15 ml) was refluxed for 0.5 h. Afterwards methanamine (0.45 g, 0.01 mol) was added and the mixture was stirred for 10 min at room temperature. The solution was kept at room temperature for 5 d. On solvent evaporation colourless single crystals of the title compound were obtained that are suitable for X-ray analysis.

### Refinement

H atoms were initially located from difference maps and then refined in a riding model with C—H = 0.96–0.97 Å, N—H = 0.89 Å, O—H = 0.82Å and *U*_iso_(H) = 1.2*U*_eq_(C) or 1.5*U*_eq_(O, N, methyl C).

### Crystal data

**Table:**

CH_6_N^+^·C_9_H_3_Cl_4_O_4_^−^	*F*(000) = 704
*M**_r_* = 348.98	*D*_x_ = 1.683 Mg m^−^^3^
Monoclinic, *P*2_1_/*c*	Mo *K*α radiation, λ = 0.71073 Å
*a* = 14.3138 (13) Å	Cell parameters from 2427 reflections
*b* = 14.2231 (14) Å	θ = 2.9–27.9°
*c* = 6.7648 (7) Å	µ = 0.87 mm^−^^1^
β = 91.021 (1)°	*T* = 298 K
*V* = 1377.0 (2) Å^3^	Block, colorless
*Z* = 4	0.45 × 0.40 × 0.38 mm

### Data collection

**Table:**

Bruker SMART CCD area-detector diffractometer	2413 independent reflections
Radiation source: fine-focus sealed tube	1752 reflections with *I* > 2σ(*I*)
graphite	*R*_int_ = 0.034
phi and ω scans	θ_max_ = 25.0°, θ_min_ = 2.9°
Absorption correction: multi-scan (*SADABS*; Bruker, 1997)	*h* = −10→17
*T*_min_ = 0.697, *T*_max_ = 0.734	*k* = −16→15
6756 measured reflections	*l* = −8→7

### Refinement

**Table:**

Refinement on *F*^2^	Secondary atom site location: difference Fourier map
Least-squares matrix: full	Hydrogen site location: inferred from neighbouring sites
*R*[*F*^2^ > 2σ(*F*^2^)] = 0.034	H-atom parameters constrained
*wR*(*F*^2^) = 0.092	*w* = 1/[σ^2^(*F*_o_^2^) + (0.036*P*)^2^ + 0.764*P*] where *P* = (*F*_o_^2^ + 2*F*_c_^2^)/3
*S* = 1.06	(Δ/σ)_max_ = 0.001
2413 reflections	Δρ_max_ = 0.27 e Å^−^^3^
176 parameters	Δρ_min_ = −0.26 e Å^−^^3^
0 restraints	Extinction correction: *SHELXL97* (Sheldrick, 2008), Fc^*^=kFc[1+0.001xFc^2^λ^3^/sin(2θ)]^-1/4^
Primary atom site location: structure-invariant direct methods	Extinction coefficient: 0.0169 (12)

### Special details

**Table:**

Geometry. All e.s.d.'s (except the e.s.d. in the dihedral angle between two l.s. planes) are estimated using the full covariance matrix. The cell e.s.d.'s are taken into account individually in the estimation of e.s.d.'s in distances, angles and torsion angles; correlations between e.s.d.'s in cell parameters are only used when they are defined by crystal symmetry. An approximate (isotropic) treatment of cell e.s.d.'s is used for estimating e.s.d.'s involving l.s. planes.
Refinement. Refinement of *F*^2^ against ALL reflections. The weighted *R*-factor *wR* and goodness of fit *S* are based on *F*^2^, conventional *R*-factors *R* are based on *F*, with *F* set to zero for negative *F*^2^. The threshold expression of *F*^2^ > σ(*F*^2^) is used only for calculating *R*-factors(gt) *etc*. and is not relevant to the choice of reflections for refinement. *R*-factors based on *F*^2^ are statistically about twice as large as those based on *F*, and *R*- factors based on ALL data will be even larger.

### Fractional atomic coordinates and isotropic or equivalent isotropic displacement parameters (Å^2^)

**Table:**

	*x*	*y*	*z*	*U*_iso_*/*U*_eq_	
Cl1	0.59548 (5)	0.44954 (5)	0.68418 (11)	0.0403 (2)	
Cl2	0.75122 (6)	0.31368 (5)	0.83762 (13)	0.0504 (3)	
Cl3	0.94431 (6)	0.31160 (6)	0.64018 (14)	0.0560 (3)	
Cl4	0.97935 (5)	0.43587 (5)	0.26682 (13)	0.0498 (3)	
N1	0.56810 (18)	0.82381 (16)	0.3492 (4)	0.0405 (6)	
H1A	0.5878	0.8600	0.4489	0.061*	
H1B	0.5950	0.8422	0.2382	0.061*	
H1C	0.5832	0.7642	0.3743	0.061*	
O1	0.82037 (15)	0.65000 (13)	0.1792 (3)	0.0405 (5)	
O2	0.8307 (2)	0.52896 (16)	−0.0279 (3)	0.0647 (7)	
O3	0.61656 (15)	0.57063 (15)	0.1743 (3)	0.0481 (6)	
O4	0.62782 (14)	0.64439 (13)	0.4641 (3)	0.0408 (5)	
C1	0.82003 (19)	0.5598 (2)	0.1337 (4)	0.0345 (7)	
C2	0.64751 (19)	0.58072 (18)	0.3451 (4)	0.0303 (6)	
C3	0.80417 (19)	0.50129 (17)	0.3163 (4)	0.0282 (6)	
C4	0.71960 (19)	0.50884 (17)	0.4148 (4)	0.0269 (6)	
C5	0.70398 (18)	0.44908 (17)	0.5740 (4)	0.0274 (6)	
C6	0.77229 (19)	0.38691 (18)	0.6410 (4)	0.0303 (6)	
C7	0.85805 (19)	0.38385 (18)	0.5494 (4)	0.0325 (7)	
C8	0.87294 (19)	0.43961 (18)	0.3837 (4)	0.0327 (7)	
C9	0.8303 (3)	0.7137 (2)	0.0141 (5)	0.0592 (10)	
H9A	0.8873	0.7001	−0.0528	0.089*	
H9B	0.8319	0.7773	0.0617	0.089*	
H9C	0.7783	0.7061	−0.0760	0.089*	
C10	0.4658 (2)	0.8321 (2)	0.3267 (5)	0.0543 (9)	
H10A	0.4367	0.8127	0.4468	0.082*	
H10B	0.4444	0.7928	0.2197	0.082*	
H10C	0.4495	0.8963	0.2988	0.082*	

### Atomic displacement parameters (Å^2^)

**Table:**

	*U*^11^	*U*^22^	*U*^33^	*U*^12^	*U*^13^	*U*^23^
Cl1	0.0319 (4)	0.0426 (4)	0.0470 (5)	0.0041 (3)	0.0120 (3)	0.0124 (3)
Cl2	0.0489 (5)	0.0470 (5)	0.0554 (5)	0.0046 (4)	0.0012 (4)	0.0251 (4)
Cl3	0.0397 (5)	0.0524 (5)	0.0758 (6)	0.0189 (4)	−0.0053 (4)	0.0093 (4)
Cl4	0.0323 (4)	0.0515 (5)	0.0660 (6)	0.0021 (4)	0.0166 (4)	−0.0093 (4)
N1	0.0551 (17)	0.0323 (13)	0.0341 (14)	0.0043 (12)	−0.0040 (12)	−0.0006 (11)
O1	0.0548 (14)	0.0320 (11)	0.0350 (11)	−0.0030 (10)	0.0087 (10)	0.0036 (9)
O2	0.097 (2)	0.0604 (15)	0.0369 (14)	−0.0146 (14)	0.0208 (13)	−0.0099 (11)
O3	0.0471 (13)	0.0616 (14)	0.0352 (12)	0.0050 (11)	−0.0105 (10)	0.0095 (10)
O4	0.0451 (13)	0.0313 (11)	0.0462 (13)	0.0112 (9)	0.0059 (10)	0.0040 (9)
C1	0.0297 (16)	0.0423 (17)	0.0317 (17)	−0.0031 (13)	0.0049 (13)	−0.0011 (13)
C2	0.0264 (15)	0.0295 (15)	0.0352 (17)	0.0005 (12)	0.0043 (13)	0.0089 (13)
C3	0.0310 (15)	0.0240 (13)	0.0295 (15)	−0.0035 (12)	0.0012 (12)	−0.0042 (11)
C4	0.0284 (15)	0.0263 (14)	0.0261 (14)	−0.0004 (12)	−0.0011 (12)	−0.0018 (11)
C5	0.0226 (14)	0.0283 (14)	0.0314 (15)	−0.0005 (12)	0.0022 (12)	−0.0028 (12)
C6	0.0341 (16)	0.0252 (14)	0.0315 (15)	−0.0017 (12)	−0.0027 (13)	0.0011 (11)
C7	0.0260 (15)	0.0258 (14)	0.0454 (17)	0.0046 (12)	−0.0043 (13)	−0.0050 (12)
C8	0.0296 (15)	0.0294 (14)	0.0391 (17)	−0.0011 (12)	0.0060 (13)	−0.0095 (13)
C9	0.074 (3)	0.051 (2)	0.054 (2)	−0.0026 (19)	0.0147 (19)	0.0210 (17)
C10	0.055 (2)	0.054 (2)	0.053 (2)	0.0031 (17)	−0.0051 (18)	0.0001 (16)

### Geometric parameters (Å, °)

**Table:**

Cl1—C5	1.734 (3)	C2—C4	1.521 (4)
Cl2—C6	1.720 (3)	C3—C8	1.389 (4)
Cl3—C7	1.712 (3)	C3—C4	1.396 (4)
Cl4—C8	1.729 (3)	C4—C5	1.393 (4)
N1—C10	1.475 (4)	C5—C6	1.388 (4)
N1—H1A	0.8900	C6—C7	1.386 (4)
N1—H1B	0.8900	C7—C8	1.393 (4)
N1—H1C	0.8900	C9—H9A	0.9600
O1—C1	1.319 (3)	C9—H9B	0.9600
O1—C9	1.447 (3)	C9—H9C	0.9600
O2—C1	1.191 (3)	C10—H10A	0.9600
O3—C2	1.238 (3)	C10—H10B	0.9600
O4—C2	1.247 (3)	C10—H10C	0.9600
C1—C3	1.510 (4)		
			
C10—N1—H1A	109.5	C7—C6—C5	119.9 (2)
C10—N1—H1B	109.5	C7—C6—Cl2	119.7 (2)
H1A—N1—H1B	109.5	C5—C6—Cl2	120.4 (2)
C10—N1—H1C	109.5	C6—C7—C8	119.5 (3)
H1A—N1—H1C	109.5	C6—C7—Cl3	119.8 (2)
H1B—N1—H1C	109.5	C8—C7—Cl3	120.7 (2)
C1—O1—C9	115.4 (2)	C3—C8—C7	120.4 (2)
O2—C1—O1	124.9 (3)	C3—C8—Cl4	119.6 (2)
O2—C1—C3	124.9 (3)	C7—C8—Cl4	119.9 (2)
O1—C1—C3	110.2 (2)	O1—C9—H9A	109.5
O3—C2—O4	127.2 (3)	O1—C9—H9B	109.5
O3—C2—C4	116.1 (2)	H9A—C9—H9B	109.5
O4—C2—C4	116.6 (2)	O1—C9—H9C	109.5
C8—C3—C4	120.5 (2)	H9A—C9—H9C	109.5
C8—C3—C1	120.0 (2)	H9B—C9—H9C	109.5
C4—C3—C1	119.5 (2)	N1—C10—H10A	109.5
C5—C4—C3	118.3 (2)	N1—C10—H10B	109.5
C5—C4—C2	122.1 (2)	H10A—C10—H10B	109.5
C3—C4—C2	119.5 (2)	N1—C10—H10C	109.5
C6—C5—C4	121.3 (2)	H10A—C10—H10C	109.5
C6—C5—Cl1	119.5 (2)	H10B—C10—H10C	109.5
C4—C5—Cl1	119.2 (2)		
			
C9—O1—C1—O2	3.0 (5)	C2—C4—C5—Cl1	5.4 (4)
C9—O1—C1—C3	−177.0 (3)	C4—C5—C6—C7	0.2 (4)
O2—C1—C3—C8	63.3 (4)	Cl1—C5—C6—C7	177.8 (2)
O1—C1—C3—C8	−116.7 (3)	C4—C5—C6—Cl2	−179.9 (2)
O2—C1—C3—C4	−115.9 (3)	Cl1—C5—C6—Cl2	−2.3 (3)
O1—C1—C3—C4	64.1 (3)	C5—C6—C7—C8	−3.6 (4)
C8—C3—C4—C5	−3.9 (4)	Cl2—C6—C7—C8	176.5 (2)
C1—C3—C4—C5	175.3 (2)	C5—C6—C7—Cl3	176.6 (2)
C8—C3—C4—C2	176.6 (2)	Cl2—C6—C7—Cl3	−3.3 (3)
C1—C3—C4—C2	−4.2 (4)	C4—C3—C8—C7	0.6 (4)
O3—C2—C4—C5	−118.0 (3)	C1—C3—C8—C7	−178.6 (2)
O4—C2—C4—C5	63.7 (3)	C4—C3—C8—Cl4	−176.7 (2)
O3—C2—C4—C3	61.5 (3)	C1—C3—C8—Cl4	4.1 (3)
O4—C2—C4—C3	−116.8 (3)	C6—C7—C8—C3	3.2 (4)
C3—C4—C5—C6	3.5 (4)	Cl3—C7—C8—C3	−177.0 (2)
C2—C4—C5—C6	−177.0 (2)	C6—C7—C8—Cl4	−179.5 (2)
C3—C4—C5—Cl1	−174.1 (2)	Cl3—C7—C8—Cl4	0.3 (3)

### Hydrogen-bond geometry (Å, °)

**Table:**

*D*—H···*A*	*D*—H	H···*A*	*D*···*A*	*D*—H···*A*
N1—H1A···O3^i^	0.89	1.86	2.742 (3)	173
N1—H1B···O4^ii^	0.89	1.93	2.794 (3)	163
N1—H1C···O4	0.89	1.92	2.797 (3)	170

Symmetry codes: (i) *x*, −*y*+3/2, *z*+1/2; (ii) *x*, −*y*+3/2, *z*−1/2.
